# Evaluating the Evolution of Social Networks: A Ten-Year Longitudinal Analysis of an Agricultural, Fishing and Forestry Occupational Health Research Center

**DOI:** 10.3390/ijerph182412889

**Published:** 2021-12-07

**Authors:** Melissa B. Scribani, Pamela J. Tinc, Erika E. Scott, Julie A. Sorensen, Nancy H. Tallman, Anne M. Gadomski

**Affiliations:** 1 Bassett Medical Center, Bassett Research Institute, Cooperstown, NY 13326, USA; pam.tinc@bassett.org (P.J.T.); erika.scott@bassett.org (E.E.S.); julie.sorensen@bassett.org (J.A.S.); nancy.tallman@bassett.org (N.H.T.); anne.gadomski@bassett.org (A.M.G.); 2 Northeast Center for Occupational Health and Safety in Agriculture, Forestry, Fishing, Cooperstown, NY 13326, USA

**Keywords:** social network analysis, transdisciplinary ties, research productivity

## Abstract

As part of our evaluation of the NIOSH-funded Northeast Center for Occupational Health and Safety: Agriculture, Forestry and Fishing (NEC), we present methodology, findings and the potential implications of a sequential social network analysis (SNA) conducted over ten years. Assessing the effectiveness of the center’s scientific projects was our overarching evaluation goal. The evaluation design employed SNA to (a) look at changes to the center’s network over time by visualizing relationships between center collaborators annually, (b) document collaborative ties and (c) identify particularly strong or weak areas of the network. Transdisciplinary social network criteria were applied to the SNA to examine the collaboration between center personnel, their partners and the industry groups they serve. SNA participants’ perspectives on the utility of the SNA were also summarized to assess their interest in ongoing SNA measures. Annual installments of the SNA (2011–2020) showed an expansion of the network with a 30% increase in membership from baseline, as well as an increase in total relational ties (any type of contact). SNA measures also indicated significant increases in co-publication, cross-sector and transdisciplinary ties. Overall, SNA is an effective tool in visualizing and sustaining an occupational safety and health research and outreach network. Its utility is limited by how ties are characterized, grant cycle timeframes and how SNA metrics relate to productivity.

## 1. Introduction

Research advancement depends on collaborative efforts among researchers. Prior studies have used social network analysis (SNA) to measure collaborative ties among researchers in order to understand the dynamics and characteristics of research productivity [1]. SNA allows visualization and quantitative measures of networks, specifically positions of intramural and extramural partners, their interconnectedness, and collaborative ties. These ties are characterized in different ways depending on the evaluative purpose of the SNA. For example, Petrescu-Prahova et al. [2] used multiple levels of contact (email, phone, and in-person communications, co-publications, co-presentations and outreach activities) as ties for evaluating the strength of the collaboration, and potentially the resulting productivity, of a public health network. In that study, collaboration outcomes were measured by examining six types of products: published articles, in-progress manuscripts, grant applications, tools, research projects and presentations. Using density as one measure of collaboration, the most cohesive networking was located within research projects, followed by presentations and in-progress manuscripts [2].

Social networks evolve over time as they are driven by a shared purpose, grant funding, linked activities and/or affiliations of their members [3]. Bian et al. [4] used a temporal approach to analyze the evolution of research collaborations among Clinical and Translational Science Award (CTSA) centers. This SNA demonstrated that the network was moving towards more collaboration, more transdisciplinary teamwork and less isolated units. Another study of CTSA collaboration used the nodes and edges of the connected components of annual SNAs to show that ties increased over six years and that the majority of CTSA researchers were connected either directly or indirectly [5]. Long et al. [6] also used annual SNAs of their translational research network to show the evolution of collaborative arrangements among members. This SNA showed a pattern of widespread collaboration that was interpreted as evidence of a well-functioning network. These studies show that sequential SNA can demonstrate collective impacts on networks over time. However, the estimation of the statistical power of such longitudinal analyses is plagued by some of the recognized limitations of SNA itself, i.e., network size, number of SNA surveys, missing data and participant turnover [7].

The Northeast Center for Occupational Health and Safety: Agriculture, Forestry and Fishing (NEC) is one of eleven National Institute for Occupational Safety and Health (NIOSH) Centers for Agricultural Safety and Health (AgFF). A formal, systematic evaluation of the NEC was designed to measure the effectiveness of the NEC and its six scientific projects (four extramural and two intramural in 2011–2016, and three extramural and three intramural in 2016–2020). The promotion of collaboration among both intramural and extramural partners has been one important function of the center’s leadership. Examples include the provision of networking opportunities at annual meetings, community planning initiatives (e.g., a Future Search three-day conference with commercial fishing stakeholders), and the expansion of strategic advisory groups (e.g., the fishing advisory group). In both cycles, SNA was used to measure relationships within the NEC network, both on an annual basis and longitudinally. Another AgFF center, the Central States Center for Agricultural Safety and Health (CS-CASH), used SNA to establish a baseline from which to document the AgFF Center’s network growth and collaboration [8]. Based on this SNA, CS-CASH decided to continue annual SNAs in order to monitor collaboration with external stakeholders, estimate the geographic reach of CS-CASH, and assess its influence. Another AgFF center, High Intermountain Plains Center for Agricultural Health and Safety (HICAHS), used SNA to describe its partnerships and better understand its collaborative ties [9].

The co-creation of knowledge and solutions requires team science and transdisciplinary collaboration across a broad array of stakeholders in an intellectual community [10] focused on a specific topic, such as AgFF occupational safety and health (OSH). Although the terms interdisciplinary, multidisciplinary and transdisciplinary are often used interchangeably, transdisciplinary is defined as a higher level of collaboration, as illustrated by the following quotes. “Whereas interdisciplinary collaborations are often emergent collaborations that result in piecemeal knowledge integrations, transdisciplinary collaborations are integrated and coordinated, such that a new field and integrated research perspective arises around a complex problem” [11]. Felknor et al. [12] define transdisciplinary research in OSH as: “Transdisciplinary efforts are those that cross multiple disciplines and professions and result in a broader and more holistic approach to problem-solving strategies.” Transdisciplinary ties imply that researchers are working outside the boundaries of their respective disciplines to mutually inform one another’s work. The team collaborates to better address complex systems and share potential research methods and solutions across multiple disciplines. These ties “transcend long-standing disciplinary boundaries and engage investigators, clinicians, public health experts and policymakers in highly innovative, yet tightly integrated translational initiatives” [12]. Criteria for characterizing transdisciplinary networks have been developed and include measures of diversity (network size and composition), integration and collaboration (network density), stability (degree centrality) and efficiency (centralization) [1].

In OSH, measuring collaboration across AgFF sectors (agriculture, forestry and commercial fishing) may be relevant as certain occupational hazards are common across these industry groups. Examples include toxic gas in confined spaces, the need for personal protective equipment, financial stress, sleep deprivation, machinery entanglements and musculoskeletal injuries. The identification of solutions in one industry may translate well into the design of solutions for OSH problems in the other industries. For example, a ventilation solution designed for manure pits by the agriculture sector could be adapted to mitigate toxic exposures in confined spaces on commercial fishing boats. 

The specific aims of this study were to visualize how the NEC network has evolved over the past ten years using SNA, enumerate collaborative ties, characterize the degree to which the network met transdisciplinary social network criteria and evaluate the utility of SNA from the perspective of NEC members and leaders. 

## 2. Materials and Methods

A roster of network members was developed annually with input from project investigators and NEC administrators. Roster members include consultants, research personnel, co-investigators and other collaborators, individuals serving on various NEC advisory boards and other stakeholders. This roster was incorporated into a web-based survey instrument that was emailed to each member. The survey collected information regarding the specific type(s) of contact with each roster member over the prior six months. In the first cycle, the types of contacts documented were emails/meetings (including video conference and conference call), co-publications (including abstracts, peer-reviewed papers and other articles), co-presentations (oral, poster, webinars and workshops) and external outreach activities. In the second cycle, the types of contacts were communication (emails, phone calls, meetings), co-publications (abstracts, peer-reviewed papers, other articles), technical innovations, translating research to practice, social marketing/promotion, training/education/community advocacy and cost analyses. Three installments of the survey (2018–2020) collected feedback from roster members regarding emerging issues in AgFF and solicited key topics to cover at annual meetings. Lastly, respondents were asked a series of process-related and open-ended questions regarding the survey itself. 

UCINET NetDraw software was used to create sociograms for “any contact” that portrayed the network overall, and for co-publication, that defines a specific type of collaboration over the grant cycles [13]. Density and measures of centrality were calculated using UCINET [14]. Visual inspection of the sociogram and analysis of degree centrality were also explored. Non-symmetric data such as measures of the in-degree of a vertex (node) (the number of ties received by that node) and out-degree (the number of ties initiated by that node) were analyzed [14].

Intramural and extramural ties were quantified, with in-house NEC staff defined as intramural, and researchers and collaborators from outside organizations, including advisory board members, defined as extramural. Industry sectors were defined according to the individual’s primary area of expertise and included agriculture, forestry, fishing, as well as a combined AgFF group for those working with a combination of sectors. An “other” classification included network members with a general occupational health focus that was not specific to one industry. 

The specific timeframes for the two five-year NIOSH grant cycles defined Cycle 1 and 2. Each cycle included a different set of AgFF research proposals and outreach plans; thus, partners and researchers were somewhat different from Cycle 1 to 2 and from year to year depending on changes in research foci, staffing, attrition and other factors (such as projects that entailed outreach to a specific group in one cycle but not the other). 

The distribution of disciplines represented by the NEC network was summarized over time to provide a measure of an evolving transdisciplinary approach to AgFF OSH problems. Those disciplines included policymakers; employers (including small, medium, and large enterprises); industrial hygiene; engineering; city, county and state governments; public health professionals; medicine and nursing professionals; environmental health and safety professionals; social scientists; social services and mental health and wellness/health promotion groups; risk management professionals; advocates and safety specialists [12]. 

The criteria for transdisciplinary network analyses [1] were applied to the NEC SNA to characterize its transdisciplinary work. Specifically, the concept of diversity was measured by examining the size and composition of the NEC network and demonstrated changes over time. The expansion of the network into more disciplines and sectors over time was also summarized. A further measure of network expansion was captured using network breadth [15], which measures the average distance between all nodes of a network, regardless of how many connections a node has. While smaller values of breadth are indicative of greater social cohesion (tighter groups), larger values of breadth are indicative of greater inclusion and expansion. Integration and collaboration are described by Steelman et al. [1] to include measures of cohesion, i.e., the overall network clustering coefficient is a measure of how much nodes cluster. It is calculated as the number of closed triads (i.e., groups of three nodes that are all connected). Clustering provides an indication of how quickly ideas, information and solutions are shared amongst members of the network (increased clustering is a positive indicator of information dissemination). Collaboration was further assessed using network density (defined as the number of observed ties divided by the total number of possible ties [16]) and group density, or the number of ties by members belonging to groups, including extramural partners as well as by specific sector. Stability is represented by degree centrality, i.e., the total number of connections a member has measured by taking the sum of in- and out-connections [16]. Efficiency was assessed by centralization and position of NEC members in sociograms. 

## 3. Results

### 3.1. Diversity and Expansion

The size and composition of the NEC SNA reflect its diversity. The NEC roster has grown by more than 30% since baseline. Over the 10-year period, 180 individuals were represented on at least one NEC roster, with 15 (8.3%) appearing on every single roster yearly. Those 15 individuals included NEC administration, researchers, and outreach core members; their persistence in the SNA over time reflects the stability of the NEC. As shown in Table 1, the survey response rates were consistently above the recommended level of 80% [17] over the 10-year period, ranging from a low of 81.0% in C1Y1 to above 90% in C1Y3 (93.1%) and C2Y1 (90.5%). After the initial year, the response rate did not dip below 84% for any of the following nine surveys.

The composition of the network by sector is summarized in Table 2. Over 10 years, the NEC network has become more integrated across AgFF industry groups, reflecting increased engagement from researchers and advisors from these sectors. Interaction across the AgFF sectors is also evidenced by the increased proportions of cross-sector ties and by the incorporation of multiple additional nodes (network members) from the fishing and forestry sectors (Table 2). The network breadth increased modestly for Cycle 2 compared to Cycle 1, indicating the development of a more inclusive structure. Sub-networks also emerged over time within the AgFF sectors. There was some overlap of the fishing and agriculture sectors (cross-sector ties) in C1Y4, but this had diminished by C2Y1. While cross-sector ties declined over Cycle 2, a forestry sub-network emerged due to additions of roster members from the forestry sector and the density of ties among those partners. 

Cycle 1 included a consistent representation of agriculture and fishing sectors, as well as combined AgFF (roster members working across all three sectors). In Cycle 2, forestry sector representatives were included in the SNA roster as forestry-related research increased in this funding cycle. An increase in representation of the agriculture sector was also evident in the final year of Cycle 2 due to the inclusion of several additional agricultural occupational health outreach personnel.

Among the 180 individuals included in the NEC rosters over the two funding cycles, nearly one quarter (24.4%) represented the primary discipline of “public health”. This discipline included a diverse representation of researchers (doctorate, master’s and bachelor’s levels), evaluators, public health practitioners, educators and librarians/information specialists. Further, several public health network members were cross-trained in areas of industrial hygiene, behavioral health/social sciences, medicine/nursing and OSH. Occupational health outreach was the second most common discipline (18.3%), followed by engineering (12.2%) and employers (7.2%) (Table 3). Over the data collection period, rosters included individuals not only from throughout the northeastern United States, but also from the Midwest, Pacific Northwest and Canada.

**Figure 1:**
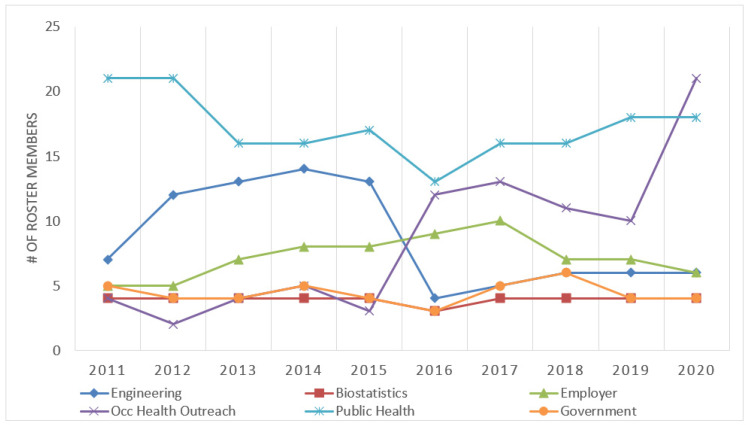
Representation of disciplines by year in the NEC SNA, 2011–2020 (not all disciplines represented due to limited sample size).

The number of social scientists and medical/nursing professionals grew in Cycle 2 compared to Cycle 1, with an average of three social scientists in Cycle 1 and six in Cycle 2, and an average of two medical/nursing professionals in Cycle 1 and five in Cycle 2. There was a marked increase in the inclusion of occupational health outreach personnel in Cycle 2, particularly in C2Y5, and a notable decrease in engineers (due to a reduced budget period for an engineering project; Figure 1). Increases in network membership in several disciplines were noted between the two cycles (not pictured). Of note, a stable number of employers were retained as part of the network over both funding cycles. Between Cycles 1 and 2, disciplines such as biostatistics, government, public health and insurance risk management had consistent representation. 

Table 4 shows the trends in peer-reviewed scientific publications and co-publication ties over the 10-year period. The ten-year average for number of peer-reviewed publications for the NEC was 9.2, ranging from five in C1Y1 to 14 in C1Y5 and C2Y3. Relating the co-publication ties (including abstracts, peer-reviewed papers, other articles) to the number of peer-reviewed publications, there were, on average, 12.3 reported co-publication ties for each published paper. Co-publication ties fluctuated over time, as have the number of abstracts and published manuscripts. This is in part due to the usual time lag between manuscript submission and acceptance for publication.

### 3.2. Integration and Collaboration

Network density (the number of ties observed, divided by the total number of possible ties in a network) decreased over the 10-year period, from a high of 0.232 in C1Y1 to a low of 0.137 in C2Y5 (Table 5). That is, in the earliest years of network measurement, nearly one-quarter of all possible connections were observed; by the end of data collection, one in seven possible connections was observed. This is a common occurrence when the network size increases, since the denominator features the number of all possible connections in a network. Network cohesion, as measured by the overall clustering coefficient, also decreased over time from a high of 0.518 in C1Y1 to 0.362 in C2Y5, indicating that network members did not form tight groups. 

The proportion of extramural ties increased, indicating that new roster members established relationships fostered by NEC collaboration. Some may have already been well-connected with each other prior to joining the NEC network (as indicated by the C1Y4 jump in extramural ties). Thus, the NEC developed important relationships that led to contacts joining the network. This reflects NEC’s ability to identify key leaders/contacts within sectors and engage them in the network. 

**Figure 2:**
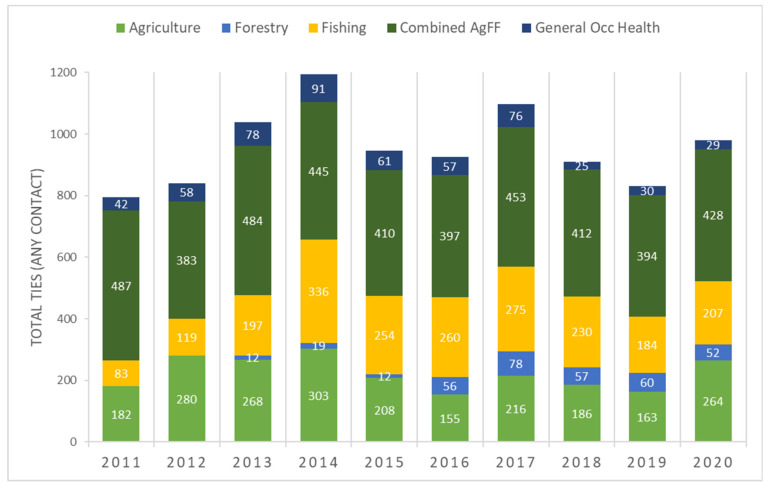
Total number of ties and distribution of ties by sector, 2011–2020.

Density by group (the number of total ties to and from members of a specific group) is used to measure collaboration. Density changed over time, with increases in the numbers of ties in specific sectors, notably in forestry in Cycle 2 due to funding for a forestry-focused scientific project (Figure 2). In the first year of data collection, the fewest extramural ties were observed and, accordingly, the highest number of sector-specific ties in the combined AgFF sector. This is because most intramural NEC staff are assigned to the combined AgFF sector. Ties in the fishing sector increased to 28.1% of all ties in C1Y4 and remained above 20% thereafter.

### 3.3. Stability 

Degree centrality (sum of a node’s direct ties) is a measure of an individual’s connectedness to others. The intramural NEC staff were highly central to the network, with median total degree centrality (sum of in- and out- connections) ranging from a low of 42.0 (in C2Y3 and C2Y5) to a high of 60.0 in C1Y4 (Table 6). Intramural staff exhibited centrality measures on the order of 2.5 to 4 times higher than extramural partners. Centrality reflects how stable the NEC has been and how well the internal NEC staff are connected both within the NEC, as well as with the center’s extramural partners.

### 3.4. Efficiency

**Figure 3:**
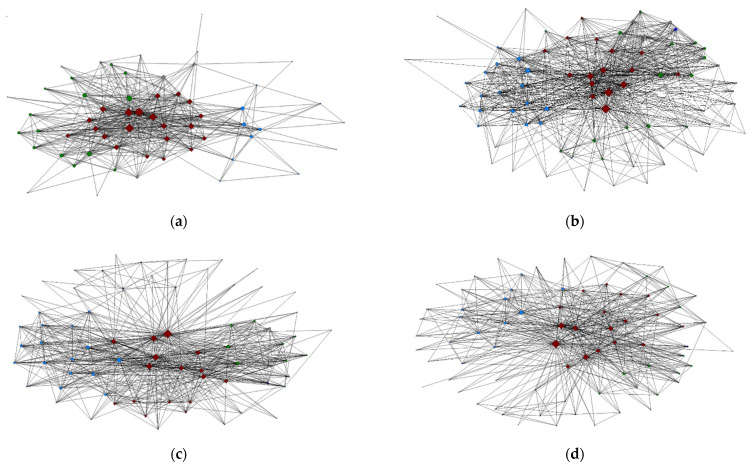
Evolution of the NEC network sociograms over grant Cycle 1 and 2. (**a**) Any contact, Cycle 1 Year 1. (**b**) Any contact, Cycle 1 Year 4. (**c**) Any contact, Cycle 2 Year 1. (**d**) Any contact, Cycle 2 Year 4. [LEGEND: Node size: centrality by degree (# of ties); Node color: Sector (Maroon = AgFF; Green = Agriculture; Light blue = Fishing; Dark blue = Forestry; Gray = Other (General Occupational Health); Node shape: Diamond = Intramural NEC staff; Circle = Extramural non-NEC].

Over the 10-year period, the NEC sociogram developed a circular, or wheel-like shape, with a denser presentation of ties towards the end of Cycle 1 (C1Y4), but not for Cycle 2 (C2Y4; Figure 3). The consistent degree centrality of internal NEC members is demonstrated by larger node sizes and development of higher degree centrality among key players in each extramural sector was noted. However, the average degree centrality of extramural partners showed a peak in C1Y4 and a slowly diminishing trend thereafter.

### 3.5. Feedback from NEC Leadership and Other Roster Members

Based on feedback from roster members, the survey itself was not considered burdensome to complete on an annual basis. Respondents reported taking 4.7 min on average to complete the survey. When asked whether the sociogram was helpful in depicting networking throughout the NEC, 65.5% of respondents said yes, citing the utility of a visual representation of the network and the ability to see the density of connections in particular sectors over time (e.g., fishing). However, when queried about the applicability of SNA to their daily work, about half (48.5%) of respondents in the C2Y5 survey noted that it was helpful. In particular, those in non-research roles (disciplines such as occupational health outreach, medicine/nursing, employers) noted that the sociograms were not relevant to their specific activities or decision-making. 

NEC leadership noted the expansion of the network into the forestry sector between Cycle 1 and Cycle 2, and the retention of forestry-sector roster members throughout Cycle 2. General mapping of “any contact” was useful for NEC administrators in terms of visualizing the most well-connected members of the network (those with the highest centrality by degree). Annual meetings of NEC researchers were key to developing and sustaining NEC relationships over time. SNA was useful in identifying outliers who the leadership felt that they should be reaching out to more or to at least try to understand why these partners were less involved. Sociograms depicting specific types of contact, such as co-publications or working together on research-to-practice activities were helpful for decision-making and planning to expand scientific collaboration. 

## 4. Discussion

This study used SNA to portray how the NEC network evolved over ten years. By enumerating collaborative ties and characterizing how the network met transdisciplinary social network criteria, SNA allowed us to visualize the extent to which the NEC fostered collaboration, diversified its membership and expanded its network to include transdisciplinary partners. 

NEC leadership reported that they used sociograms to identify areas where the network could be strengthened. For example, the lack of forestry representation in Cycle 1 led to the addition of forestry roster members and expansion over the next cycle. Measures of social cohesion decreased over time for the NEC network, as evidenced by lower network clustering coefficients and lower overall network density. However, the growth of the NEC network has become more inclusive over time, by reaching each of the occupational sectors (agriculture, forestry and fishing) and by expanding the disciplines of network members. A highly cohesive network may not be realistic or desirable for the NEC structure, as high network clustering coefficients indicate the formation of small, tight groups [1]. Evolution toward more transdisciplinary OSH centers as recommended by Felknor et al. [12] and Tamers et al. [18] necessitates diversification and expanding networks, which the NEC SNA demonstrates. However, as the number of network members increases and the network expands, network density measures tend to decrease [1]. Nevertheless, for a network that is our size, the goal of improving network density by increasing the number of observed ties out of all possible ties might further enhance collaboration in the future. 

The most critical contribution SNA could have in our evaluation is demonstrating how network collaboration (as measured by any contact) leads to tangible productivity (as measured by co-publication and co-presentation) over the grant cycles. Although these ties are associations and do not imply causation, without collaboration, there would be no co-developed products. While collaboration may not be directly causal, it is a necessary component of productivity. Is enough known about the relationship between the types of collaboration in such networks and their productivity to inform the structuring of such networks? Does the evolution of the SNA over time further our understanding of the relationship between collaboration and productivity? SNA metrics to measure whether research productivity is a function of network ties over time have not been developed yet, so future research should address how these links can be substantiated [1,19]. An additional challenge is how to define productivity, particularly productivity metrics that are important to extramural network members and workers in the AgFF community. While publications and presentations are measures of academic productivity that funding agencies value, these are not outcomes that commercial AgFF partners are likely to value. Future studies that demonstrate the links between SNA metrics and network productivity are needed.

The application of transdisciplinary criteria to this SNA suggests that the NEC network is diverse, stable, integrated and expanding over time. Despite the benefits of transdisciplinary work, there is little evidence that suggests a specific level or degree of transdisciplinary collaboration that would be required for an OSH center to be productive. This is partly compounded by the lack of a standard definition of transdisciplinary research in the literature because it is not immediately apparent how this term can be consistently applied in SNA. Going forward, we plan to carefully define disciplines in our next cycle and refine our application of the term transdisciplinary. Future research will require clear definitions and parameters of what it means to be transdisciplinary versus multidisciplinary or interdisciplinary, and how these ties influence the center’s productivity. Future research should also target how to translate SNA findings into recommendations or actions that improve transdisciplinary networks.

### Limitations 

Unlike other AgFF centers located within degree conferring institutions, the NEC is situated within a not-for-profit, rural health network in Central NY. Therefore, it has less immediate access to academic OSH specialists (e.g., industrial hygienists), fewer graduate students and less traditional educational resources that limit its comparability to other centers that are co-located within universities. Therefore, the NEC network and its evolution may be unique, and the results of this SNA may not be generalizable to other AgFF centers. The limitations of SNA include participant recall, definition of the network boundary (i.e., who to include or exclude) and five-year timeframes during which researchers may change depending on the research projects included in the grant cycle. Feedback from participants about the utility of the SNA for general understanding of the NEC network was mixed. This may be a function of different levels of understanding of SNA among these participants. A more informed approach to the use of SNA may be required to enable participants to use this information. 

## 5. Conclusions

Sequential SNA has informed our evaluation of the effectiveness of the NEC as an AgFF center. Over ten years, the NEC network expanded and became more diverse, developed collaboration across agriculture, forestry and fishing sectors, comprised a well-connected central structure and maintained engagement of network members. Overall, SNA is an effective tool in visualizing and sustaining occupational safety and health research and outreach networks. Next steps for investigation include optimizing definitions of transdisciplinary collaboration and assessing how SNA metrics relate to or reflect center productivity.

## Figures and Tables

**Table 1:** Summary of roster sizes, survey response rates and degree centrality measures over the ten-year study period.

	C1Y1	C1Y2	C1Y3	C1Y4	C1Y5	C2Y1	C2Y2	C2Y3	C2Y4	C2Y5
Data collection year	2011	2012	2013	2014	2015	2016	2017	2018	2019	2020
Total roster members	59	61	71	76	74	72	84	76	76	85
Survey response rate (%)	81.0	88.5	93.1	85.7	87.8	90.3	86.6	84.2	84.2	85.9

**Table 2:** Numbers of roster members by year and sector.

	C1Y1	C1Y2	C1Y3	C1Y4	C1Y5	C2Y1	C2Y2	C2Y3	C2Y4	C2Y5
Total Roster Members	59	61	71	76	74	72	84	76	76	85
Network Breadth	0.520	0.474	0.479	0.491	0.512	0.510	0.538	0.552	0.546	0.536
Members by Sector										
Agriculture	18	24	25	26	23	19	25	24	20	32
Forestry	0	0	1	1	1	11	11	9	11	10
Fishing	14	13	20	26	26	20	22	18	20	19
Combined AgFF	21	18	18	16	16	16	18	19	18	18
Other	6	6	7	7	8	6	8	6	7	6

**Table 3:** Distribution of disciplines combined across ten years of SNA surveys, 2011–2020 (*n* = 180 individual roster members).

Roster Member’s Main Discipline	Frequency	Percent
Public Health	44	24.4
Occupational Health Outreach	33	18.3
Engineering	22	12.2
Employer	13	7.2
Government	11	6.1
Social Scientist	10	5.6
Media/Promotions	6	3.3
Advocacy	5	2.8
Insurance (Risk Management)	5	2.8
Medical/Nursing	5	2.8
Biostatistics	4	2.2
Safety Specialist	4	2.2
Economist	3	1.7
Environmental Health and Safety	3	1.7
Government (Enforcement)	2	1.1
Management	2	1.1
Wellness Promotion	2	1.1
Industrial Hygiene	1	0.5
Marine Biologist	1	0.5
Professional Associations	1	0.5
Social Services	1	0.5
Veterinarian	1	0.5
Workforce Specialist/Management	1	0.5

**Table 4:** Number of publication-related ties in the NEC SNA over ten years.

	C1Y1	C1Y2	C1Y3	C1Y4	C1Y5	C2Y1	C2Y2	C2Y3	C2Y4	C2Y5	10-Year Mean
Co-Publication Ties	83	89	70	84	122	121	89	99	107	138	100.2
Number of peer-reviewed publications during calendar year	5	6	10	11	14	7	8	14	11	6	9.2
Ties per peer-reviewed publication	16.6	14.8	7.0	7.6	8.7	17.3	11.1	7.1	9.7	23.0	12.3

**Table 5:** Indicators of integration and collaboration of the NEC network over ten years.

	C1Y1	C1Y2	C1Y3	C1Y4	C1Y5	C2Y1	C2Y2	C2Y3	C2Y4	C2Y5
Network Clustering Coefficient	0.518	0.428	0.430	0.454	0.403	0.422	0.363	0.379	0.359	0.362
Network density	0.232	0.230	0.209	0.209	0.175	0.181	0.157	0.160	0.146	0.137
Total ties (any contact)	794	840	1039	1194	945	925	1098	910	831	980
Extramural ties (extramural to extramural)	129	191	243	388	287	250	329	225	178	275
% of total extramural ties	16.2	22.7	23.4	32.5	30.4	27.0	30.0	24.7	21.4	28.1

**Table 6:** Degree centrality measures of the NEC network over ten years (IQR = interquartile range).

	C1Y1	C1Y2	C1Y3	C1Y4	C1Y5	C2Y1	C2Y2	C2Y3	C2Y4	C2Y5
Median degree centrality total (IQR)	25.0 (9–40)	22.0 (13–36)	25.0 (11–38.5)	30.0 (11.8–43.4)	19.5 (9–34)	21.0 (9.5–34.8)	19.5 (10–32)	17.0 (8–33.3)	14.0 (7–29.3)	15.0 (8–32)
Median (IQR) intramural	47.0 (40–52)	46.0 (36–63)	49.0 (43.3–70.8)	60.0 (43.8–81.8)	49.5 (33.8–69)	51.5 (34–73)	44.0 (32–93)	42.0 (34.3–93)	44.0 (31.3–67)	42.0 (32–56)
Median (IQR) extramural	12.0 (5–23.8)	16.5 (10.8–24.3)	18.0 (10–29)	18.5 (10–35.3)	14.5 (7.3–30)	20.0 (5.8–25.3)	17.0 (9.5–25)	10.5 (6–22.3)	10.5 (6–17.3)	10.0 (6.8–19.3)

## Data Availability

The de-identified data presented in this study are available on request from the corresponding author.
